# Ergonomic Mechanical Design and Assessment of a Waist Assist Exoskeleton for Reducing Lumbar Loads During Lifting Task

**DOI:** 10.3390/mi10070463

**Published:** 2019-07-10

**Authors:** Xu Yong, Zefeng Yan, Can Wang, Chao Wang, Nan Li, Xinyu Wu

**Affiliations:** 1 Key Laboratory of Human-Manchine-Intelligence Synergic Systems, Shenzhen Institutes of Advanced Technology, Chinese Academy of Science (CAS), Shenzhen 518055, China; 2 Guangdong Provincial Key Lab of Robotics and Intelligent System, Shenzhen Institutes of Advanced Technology, Chinese Academy of Science (CAS), Shenzhen 518055, China; 3 Department of Mechanical Engineering and Intelligent Systems, The University of Electro-Communications, 1-5-1 Chofugaoka, Chofu, Tokyo 182-8585, Japan; 4 School of Mechanical Science & Engineering, Huazhong University of Science and Technology, Wuhan 430074, China

**Keywords:** waist exoskeleton, muscular activity, IEMG, lifting task

## Abstract

The purpose of this study was to develop a wearable waist exoskeleton to provide back support for industrial workers during repetitive lifting tasks and to assess reductions in back muscular activity. The ergonomic mechanical structure is convenient to employ in different applications. The exoskeleton attaches to the wearer’s body with 4 straps, takes only 30 s to put the exoskeleton on without additional help, weighs just 5 kg and is easy to carry. The mechanical clutch can assist the wearer as needed. Inertia Measurement Unit (IMU) was used to detect wearers’ motion intention. Ten subjects participated in the trial. Lower back muscle integrated electromyography (IEMG) of the left and right lumbar erector spinae (LES), thoracic erector spinae (TES), latissimus dorsi (LD) were compared in symmetrical lifting for six different objects (0, 5, 10, 15, 20, 25 kg) under two conditions of with and without the exoskeleton. The exoskeleton significantly reduced the back muscular activity during repetitive lifting tasks. The average integrated electromyography reductions were 34.0%, 33.9% and 24.1% for LES, TES and LD respectively. The exoskeleton can reduce burden and the incidence of strain on lumbar muscles during long-term lifting work.

## 1. Introduction

Despite the widespread use of robots and work-related automation equipment in modern factories to lift loads that are heavy and beyond human ability, there are still many tasks that require manual operation, especially in the logistics, manufacturing, and medical industries. According to the National Bureau of Statistics of China survey, there is an enormous and growing number of workers doing manual handling every year [1]. Long-term heavy lifting activities can significantly increase the lumbar spine and waist injuries. According to the survey, low back pain is the main problem suffered by heavy lifting workers. The proportion of workers who work in logistics and suffer from back pain is 84%, the proportion of workers who engage in construction and suffer from lumbar spine compression is 75%, and the proportion in medical care industry is 67%. In addition, there are about 31% to 45% of workers doing heavy physical labor retire due to muscle and tendon injuries [2]. The resulting economic compensation for work-related diseases and individual lumbar spine injuries not only affect the workers’ quality of life, increase the economic costs of enterprises, but it is also a major societal problem [3] with the increased number of patients with lumbar muscle strain, there is an urgent need to develop a portable and lightweight lumbar exoskeleton aimed at providing assistive torque to users who do heavy or repetitive lifting tasks.

Nowadays, many universities and research institutes are developing powered assist devices to protect the lower back of workers who do frequent manual handling and lifting tasks whether in manufacturing, construction, meatpacking or nursing care. All kinds of lower back assist exoskeletons have been designed to provide assistance between the torso and thighs to reduce lumbar spinal loading. These exoskeletons can be divided into active and passive ones according to the actuator.

Various passive exoskeletons have been proposed, such as Personal Lifting Assistive Device (PLAD) [4,5], ’Wearable moment restoring device’ [6], Bending non-demand return (BNDR) [7], Happyback [8], Bendezy [8], The PLAD uses elastic straps to provide mechanical assistance for lifting and lowering objects. The Wearable moment restoring device uses a spring to store energy when wearers bend forward, and while in same position, the stored energy supports wearers to keep that posture or to erect the trunk. The Happyback is also a passive back support device, which use bungee cords to provide assistance when wearers are doing stooped work. Erector spinae activity was reduced 23% when static holding of 0, 4 and 9 kg with the Happyback [8]. The BNDR also uses springs to provide mechanical assist for users during stooped working. For the BNDR, a reduction in back muscle force was also found in lifting tasks, but reductions in waist muscle activity were due to the exoskeleton’s ability to limit torso flexion [8]. A recent study [9] proposed a powerless waist assist device using a torsion spring. It can store gravitational potential energy in the mechanical spring when wearers bend forward, and the stored energy is released when carrying or lifting an object. The significant advantage of this device is that it is lightweight and portable, but the force provided by the device is inadequate.

Various commercial active exoskeletons have been also designed, such as the ATOUN Model A, the ATOUN Model AS, ATOUN Model Y [10], HAL Lumbar [11], Muscle Suit [10] and WSAD [12]. The ATOUN Model Y was designed to provide lower back support during lifting and holding tasks. It automatically estimates the user’s movement motion and erects users’ torso while pushing on their thighs. The physical load on lower back was reduced by 15 kg. The Muscle Suit uses an artificial muscle as the actuator to assist wearers lifting and static holding loads up to 35 kg [10]. Sasaki et al. [13] proposed a wearable lumbar assist device. The device has many more degrees of freedom that can satisfy various activities of the human body, which is mainly used for assistance by medical personnel. Other studies [14] have introduced a wearable stoop assist device which can decrease waist erector spinae activity during stooped work. This type of equipment is mainly controlled by two tension bands. Two tension bands extend from the chest strap to corresponding pulleys which are fixed to the respective output shafts of the servo motor. Muramatsu et al. [15] developed an exoskeleton that can reduce lumbar load and back muscle activity. It needs to be worn over the entire body and takes a long time to put on, and is not portable and expensive. A wearable powered assist exoskeleton for lower back support has been proposed [16]. It is convenient for wearers to work with in different environments, such as in a small room and on stairs. The total weight of the system is 6.5 kg including a battery, a control board and frames. A lower-back exoskeleton for back support has also been developed [17], which provides assistance to workers when lifting or lowering heavy objects. A series-elastic was employed on the hip joint modules, and an admittance control strategy was adopted. The weight of the whole device is 11.2 kg, and the ankle and knee joints are passive. This exoskeleton is limited by the weight of the system.

To address above issue, we present a lightweight, wearable powered lumbar exoskeleton designed to provide assistance to the human body during lifting tasks, thereby reducing lumbar spine compression to lower the risk of developing Worked-Related Musculoskeletal Disorders (WMSDs) of the users’ back. We optimized the exoskeleton’s mechanical structures to make the device convenient to use with many applications. Optimal dimensions were implemented in the mechanical design to imitate human lumbar vertebrae joints. The weight of the device needed to be light since workers will wear the equipment for extended durations. It becomes a burden when users have to wear a heavy exoskeleton for a long time [18]. The total exoskeleton system weighs just 5 kg and is easy to carry. To provide effective assistance, we used an actuator with a continuous torque of 64 N·m on both hip joints to actively assist hip flexion/extension. The exoskeleton attaches to the wearer’s body with 4 straps, and users require only 30 s to put the exoskeleton on without additional help. The mechanical clutch plays an important role in the driven module of the exoskeleton, with it the exoskeleton can assist as needed, where the motors is used only when the wearer requires assistance. This ensures that the power consumption is maintained within a relatively low level and dramatically improves the charge cycle length. We collected lower back muscles’ electromyography (EMG) signal for evaluating the effectiveness of the assistance from the device, and the wearer’s movement intention was estimated by the inertial measurement unit (IMU). A comparison of identified industrial exoskeletons with the SIAT (Shenzhen Institutes of Advanced Technology) waist exoskeleton (exoskeleton presented in this paper) shown in Table 1.

We describe the lumbar structure and lumbar load in Section 2. Mechanical structure design and hardware description of the exoskeleton are presented in Section 3. The experimental produce is shown in Section 4. The experimental results and analysis are exhibited in Section 5, and conclusions and future work are discussed in Section 6.

## 2. Biomechanical Analysis

### 2.1. Lumbar Structure

**Figure 1:**
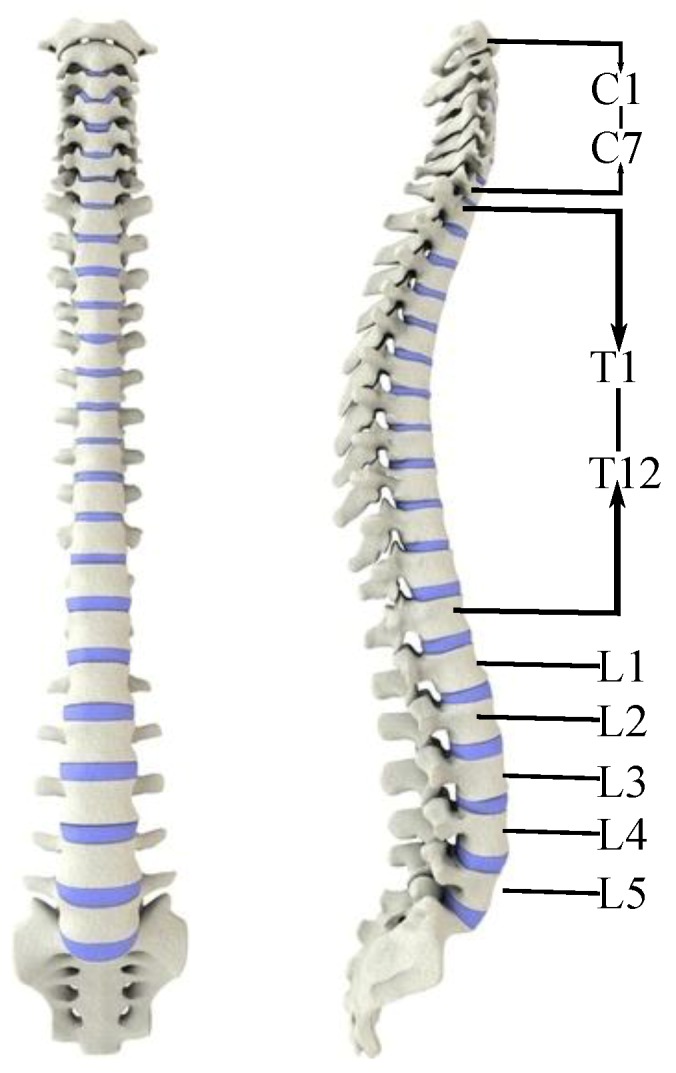
The human vertebra.

The spine is located in the middle of the posterior wall of the torso and consists of 33 vertebrae [19]. The spine functions to support weight, provide motion and protect internal organs. Vertebrae are usually classified as cervical, thoracic, lumbar, sacral, and coccyx. The cervical spine consists of seven vertebrae represented as C1–C7. The thoracic vertebra has twelve vertebrae, T1–T12, and the lumbar vertebrae is made up of five vertebrae, L1–L5 as shown in Figure 1. The sacrum is composed of five vertebrae, S1–S5. From the front, the vertebral body is gradually widened from top to bottom, and the second sacral vertebrae is the widest. It can be seen from the side that the spine has the shape of S, denoted cervical lordosis, thoracic kyphosis, lumbar lordosis and sacral kyphosis [19].

The upper limb is connected to the spine by means of the humerus, clavicle and sternum, and the lower limb is connected to the spine by the pelvis. The various activities of the upper and lower limbs are adjusted through the spine to maintain body balance [19]. In addition to support and protection, the spine also has a flexible movement function. Although the range of motion between adjacent two vertebrae is small, when multiple vertebrae actions are accumulated, they can perform a wide range of motion, such as flexion and extension, lateral flexion and rotation. However, lumbar flexion and extension are often associated with disc pressure, which can lead to varying degrees of low back pain [19].

### 2.2. Lumbar Load

**Figure 2:**
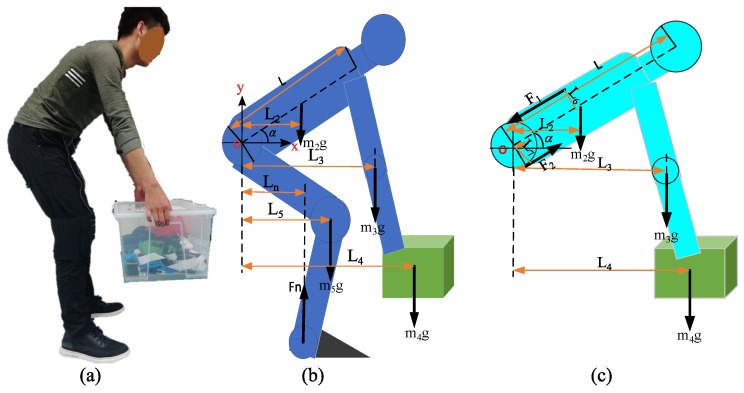
The figures: (**a**) subject lifting heavy objects, (**b**) simplified human body model, (**c**) human upper body force model.

Figure 2b is a simplified model of the human body carrying a heavy object. $m_{2}$ is the mass of trunk part and head of human body. The human’s upper limbs and hands are simplified as one unit. $m_{3}$ is the mass of human arm and hand. $m_{4}$ is object’s mass. $m_{5}$ is the mass of lower limbs of human body. $F_{n}$ is the ground support force for human body.

Compare all the forces to the o point, based on the static force balance:

(1) $$\left(m_{2}L_{2}+m_{3}L_{3}+m_{4}L_{4}+m_{5}L_{5}\right)g=F_{n}L_{n}$$

The simplified model of human upper body lifting objects is intercepted for the force exerted by the waist muscle analysis shown in Figure 2c. $F_{1}$ is the tensile force of the waist muscles, and $F_{2}$ is the support force of the abdomen and hip joints, following static equilibrium conditions:

(2) $$F_{1}L_{6}+F_{2}L_{7}-\left(m_{2}L_{2}+m_{3}L_{3}+m_{4}L_{4}\right)g=0$$

(3) $$F_{1}=\frac{\left(m_{2}L_{2}+m_{3}L_{3}+m_{4}L_{4}\right)g-F_{2}L_{7}}{L_{6}}$$

Assuming that the body mass is *m*, according to prior research [10], $m_{3}=0.044m$, $m_{2}=0.386m$, abdominal and hip support $F_{2}=0.12$ mg. The distance from the center of the skull to the center of the waist is denoted L, then $L_{4}=L\cos\alpha$, $L_{3}=0.82L\cos\alpha$, $L_{2}=0.43L\cos\alpha$, $L_{7}=0.15L\cos\alpha$, Substituting Formula (3):

(4) $$F_{1}=\frac{(m_{4}+0.23m)gL\cos\alpha }{L_{6}}$$

The value of $L_{6}$ is 3–5 cm [20]. When the weight of the object is 20 kg, the mass of the human body is 60 kg, the bending angle is 25${}^{\circ }$ and the *L* is about 0.6 m. The tensile force of the waist is 2839 N–4732 N. When the mass of the lifted object increases, these two forces will continue to increase, and back muscles will withstand a large pulling force, which will cause certain damage to the back muscles and easily cause back pain of workers for a long time. The application of an external force can effectively alleviate the tensile force on the back muscles.

## 3. Exoskeleton System Design

### 3.1. Design Theory Analysis

**Figure 3:**
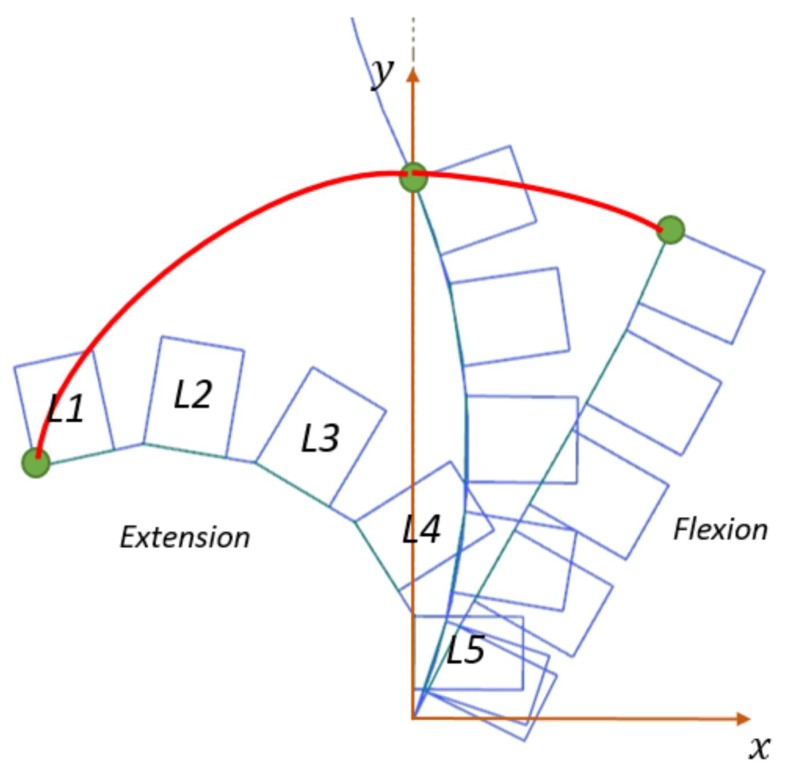
The flexion/extension curve of lumbar joints.

The exoskeleton provides assistance in the sagittal plane. We only evaluated the lumbar vertebrae joint changes in the sagittal plane. The lumbar vertebrae comprise five vertebrae represented L1 to L5. There is a certain degree of deviation during bending and ascending due to the different sizes of the exoskeleton and lumbar joints, which affects the comfort level of the waist assist exoskeleton [21]. Therefore, it is necessary to optimize the model to gain the appropriate size of the lumbar assist device. When adults stand upright, the bending radius of the lumbar joint curve is approximately 19 to 24 cm [9]. The lumbar joint on account of the sagittal plan size of the L1 to L5 is shown in Figure 3.

The intersection points of L5 of the lumbar vertebral and the sacrum are selected as the origin points to establish the rectangular coordinate system. The rotation angle of lumbar vertebra L1 to L5 served as the design parameter [5], and the lumbar vertebra joint motion curve in extreme flexion and complete extension can be determined. In the current coordinate system, the coordinates of lumbar vertebrae L1 of the motion curve in extreme flexion is (81.75 mm, 154.6 mm), in the upright position (0 mm, 172.4 mm), and in complete extension (−121.14 mm, 80.11 mm), respectively. The moving radius of overall lumbar vertebrae joints are calculated by the least square’s method:

(5) $$\sum_{i=1}^{5}{\left({x_{i}}^{2}+{y_{i}}^{2}+Ax_{i}+By_{i}+C\right)}^{2}=0.$$

Solving the partial differential equations (PDE) in terms of A, B and C as follows:

(6) $$\begin{array}{c} \frac{\partial }{\partial A}=A\sum {x_{i}}^{2}+B\sum x_{i}y_{i}+C\sum x_{i}+\sum x_{i}^{3}+\sum x_{i}y_{i}^{2}=0\\ \frac{\partial }{\partial B}=A\sum x_{i}y_{i}+B\sum y_{i}^{2}+C\sum y_{i}+\sum x_{i}^{2}y_{i}+\sum y_{i}^{3}=0\\ \frac{\partial }{\partial C}=A\sum x_{i}+B\sum y_{i}+C\sum 1+\sum x_{i}^{2}+\sum y_{i}^{2}=0. \end{array}$$

From the equations above, the coordinate values of the circle center are (16.7 mm, 17.65 mm), and the circle radius is 144.6 mm. The approximate length from the gyration center of the hip joint to the lumbar vertebrae L1 terminal is r, The projection of the center on the Y-axis and the length L5 of the tibia is about 254 mm.

### 3.2. Overall Mechanical Structure

**Figure 4:**
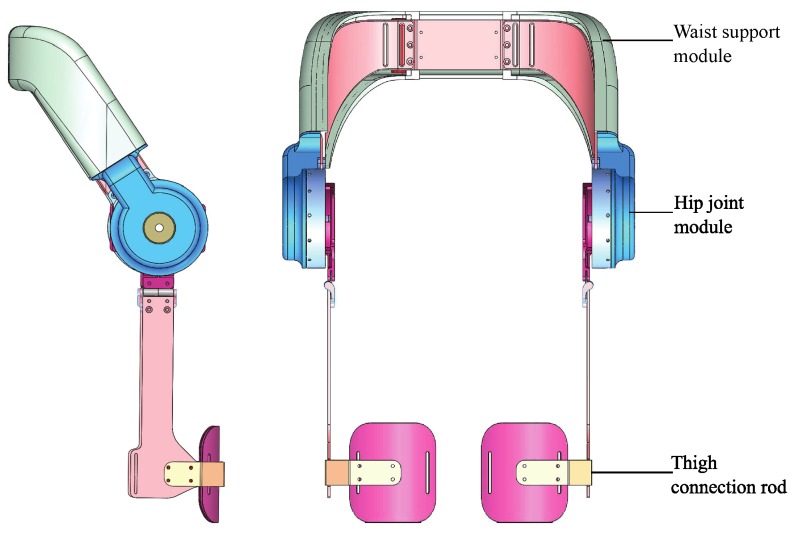
The front and side view of the overall mechanical structure.

The overall mechanical structure of the exoskeleton is shown in Figure 4, which includes a waist support module, a hip joint module and a thigh connecting rod. The waist support structure has a concave shape, which fits wearers lower-back better with its curved surface. To optimize the mechanical mechanism of the device, we placed the battery system, the electrical hardware system and motor driver in the waist support module. It is important that the weight of the device should be light because workers will wearer the equipment for extended periods. It is a burden when users have to wear a heavy exoskeleton for an extended period. The weight of the wearable waist assist exoskeleton is 5 kg and comfortable to users over a wide range of applications. To provide effective assist torque, we use an actuator module with a continuous torque of 64 N·m on both hip joint to assist hip flexion/extension. The thigh link can be rotated relative to the hip joint module, backward by $25^{\circ }$, and forward by $130^{\circ }$, which can meet the typical walking requirements of the human body. The hip joint can assist abduction/adduction motion. An adjustable mechanism is designed for altering the waistline size to accommodate different wearers. There is a leg bandage and a thigh baffle on the thigh attached the exoskeleton to the human leg. The exoskeleton involves only 4 straps to put on, users require 30 s to put on without assistance. The thigh baffle can increase the force area to reduce injury to the thigh when wearers bend.

**Figure 5:**
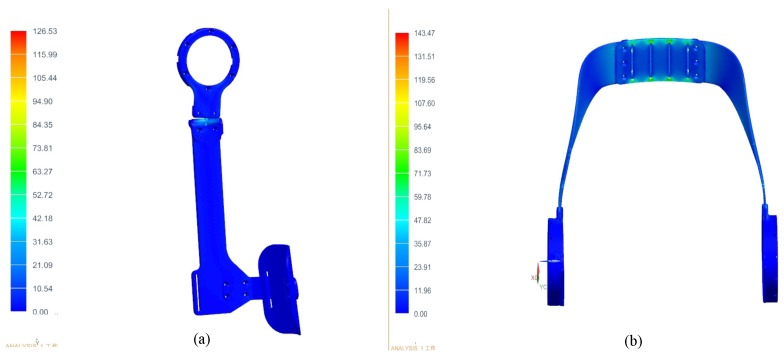
The result of FEA on thigh link and waist support board. (**a**) thigh link, (**b**) waist support board.

The thigh link and waist support board are directly connected with the motor, the moment arm at the end of the exoskeleton is long when the motors are working, and bending deformation could occur in these areas. Finite Element Analysis (FEA) is applied to these parts to ensure safety, the load applied to one end of thigh link is 64 N·m, the other end of thigh link is fixed, and the results are shown in Figure 5a,b. The maximum stress of the thigh is 126.53 MPa, and the maximum stress of the waist support board is 143.47 MPa.

### 3.3. Hip Joint Module

**Figure 6:**
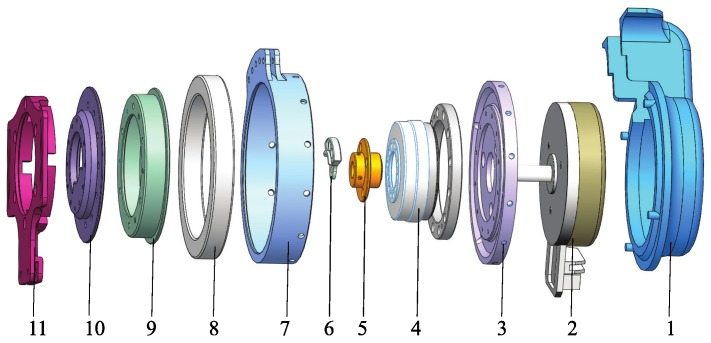
Mechanical design of hip joint. 1—shell, 2—motor, 3—flange, 4—Harmonic Drive, 5—coupling, 6—clutch, 7—bearing outer bezel, 8—bearing, 9—bearing inner bezel, 10—end cap, 11—link.

The wearable waist assist exoskeleton designed in this paper has active flexion/extension on both hip joints shown in Figure 6, which is driven by a flat, brushless motor (EC90, Maxton motor) and harmonic drive gear (Harmonic Drive, Japan hip module with a maximum torque of 64 N·m. The mechanical clutches are not powered. The clutches clock joints when the wearer turns to upright from a stooped posture while the motors start working. The clutches unlock the joints when assistance is not required and free the wearer’s movement. The motors are dormant until the next operation begins. With this approach, the power consumption is low and the clutch greatly increases the battery powered run time.

**Figure 7:**
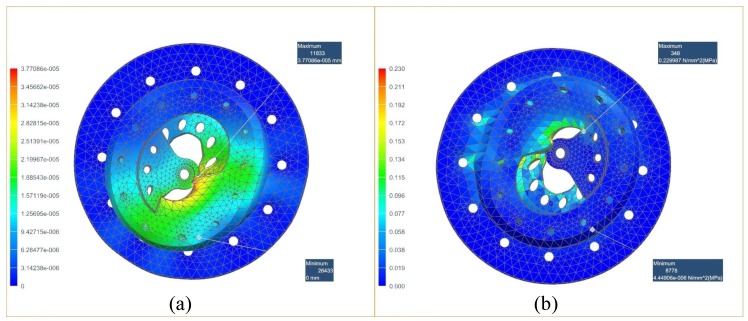
The result of FEA on mechanical clutch. (**a**) total deformation, (**b**) equivalent stress.

FEA was used to optimize the structure and ensure that the clutch remains in its elastic zone. By analyzing the Formula (4), we can see that the force applied to human body is greatest when the user initiates a lift. When the weight of the object is 30 kg, the mass of the human body is 60 kg, the bending angle is 0${}^{\circ }$ and the *L* and the L is about 0.6 m. The assist ratio is 0.5, and the force provided by the exoskeleton is approximately 4014 N. The mechanical clutch is made of aluminum 6061, and the result of FEA is shown in Figure 7. The maximum deformation of the mechanical clutch is $3.77\times 10^{-5}$ mm and the maximum stress is 0.229987 MPa, which are less than the allowed value for aluminum.

**Figure 8:**
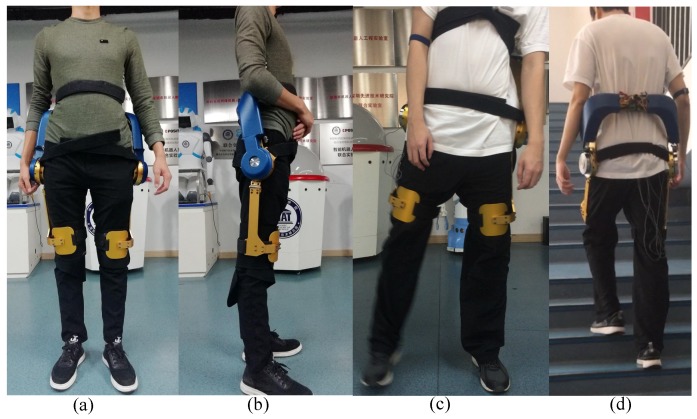
The subject wearing the exoskeleton and performing different activities. The (**a**) front and (**b**) side view, the (**c**) front view with a hip abduction, the (**d**) back view with ascending stair.

Figure 8 shows the wearer wearing the waist assist exoskeleton and demonstrating different postured of normal daily activities. The front view in Figure 8a, a side view in Figure 8b, a hip abduction in Figure 8c, the back view with ascending stair in Figure 8d. The SIAT waist exoskeleton can reduce the strain of the erector spinae and the latissimus dorsi when lifting objects, which is beneficial to workers who lift heavy loads.

### 3.4. Embedded Electronics

**Figure 9:**
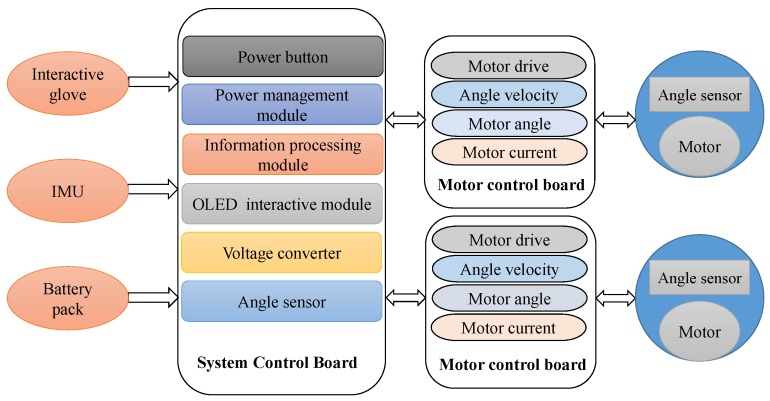
The electronic system with powered exoskeleton.

The electrical hardware system of the device is shown in Figure 9, which consists of motor units, motor control board, system control board and the extended device module. Each joint has one motor with a voltage of 24 V and power of 90 W, and incremental encoder (6400 pulse repetition, Maxon Motor). Motor control board has a motor driver (Accelnet APM-090-30) and three sensors, which can record the motor parameters of angle, angular velocity and current in real time. An OLED interactive module is used for the power management module and is integrated into the system control board of the exoskeletons. The battery management module is the fundamental unit of the operating system. To satisfy the requirements of the usage scenarios and working hours, the battery consists of 18,650 lithium battery units placed in the waist support structure [17].

### 3.5. Dynamical Model Analysis

**Figure 10:**
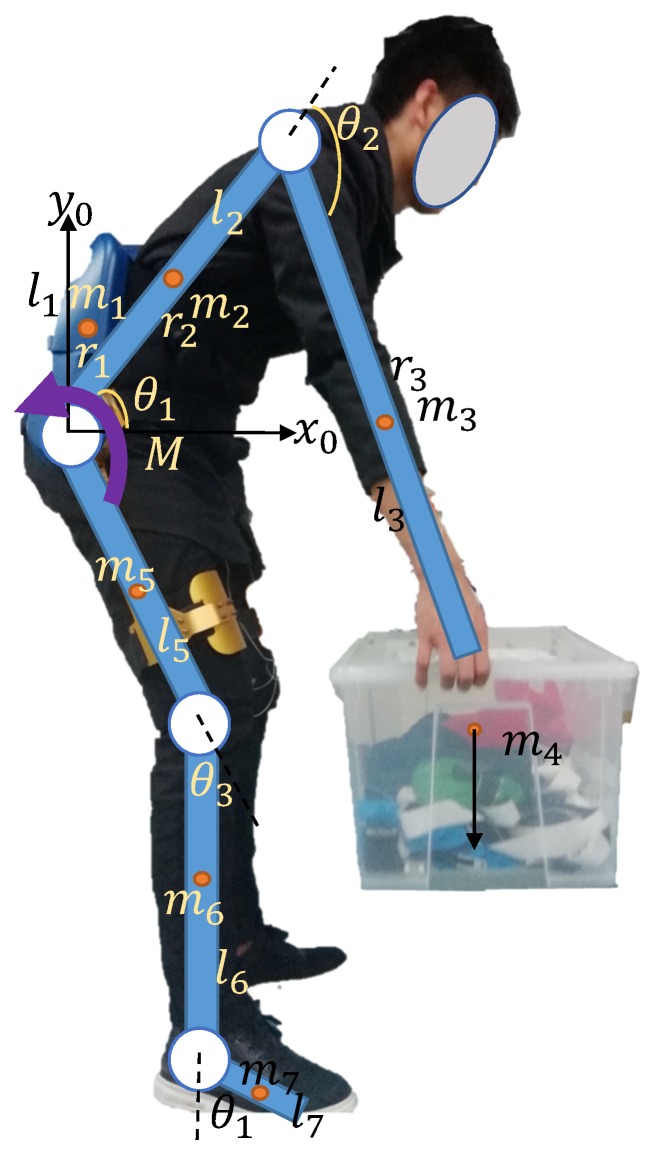
The human-exoskeleton coupling dynamics model.

To obtain the exoskeleton dynamic system characteristics and then accurately control the exoskeleton motion, the human-exoskeleton coupling dynamics model is established in this paper. The system dynamics model is established by the Euler Lagrange equation method [22]. As shown in Figure 10, $m_{1}$ is the mass of waist module of the exoskeleton. $l_{1}$ is the length of the exoskeleton. $m_{2}$ is the mass of the trunk part below the neck, the neck and head, $l_{2}$ is the length of the trunk. The human upper limbs, arms and hands are simplified, $m_{3}$ is the mass of the human arm and hand, $l_{3}$ is the length of the upper limb. $m_{4}$ is the mass of the objects. $m_{5}$ is the mass of the thigh and the $l_{5}$ is the length of the thigh. $m_{6}$ is the mass of shank, the $l_{6}$ is the length of the shank. $m_{7}$ is the foot mass and the $l_{7}$ is the foot length. 0 point selected as the origin to establish an x-y Cartesian coordinate system.

The transformation matrix from coordinate system *i* to coordinate system *i*−1 is [17]:

$${}_{i}^{i-1}T=\left[\begin{array}{cccc} c\theta_{i} & -s\theta_{i} & 0 & \alpha_{i-1}\\ s\theta_{i}c\alpha_{i-1} & c\theta_{i}c\alpha_{i-1} & -s\alpha_{i-1} & -s\alpha_{i-1}d_{i}\\ s\theta_{i}s\alpha_{i-1} & c\theta_{i}c\alpha_{i-1} & c\alpha_{i-1} & c\alpha_{i-1}d_{i}\\ 0 & 0 & 0 & 1 \end{array}\right]$$

The transformation from coordinate system *N* to coordinate system 0 is [17]:

(7) $${}_{N}^{0}T={}_{1}^{0}T{}_{2}^{1}T{}_{3}^{2}T\cdots {}_{N}^{N-1}T$$

The centroid position vector, the centroid velocity vector and the centroid angular velocity vector of the exoskeleton are:

$${}_{1}^{0}\vec{P}=\left[\begin{array}{c} r_{1}c_{1}\\ r_{1}s_{1}\\ 0 \end{array}\right]{}_{1}^{0}\vec{V}=\left[\begin{array}{c} -r_{1}s_{1}{\dot{\theta }}_{1}\\ r_{1}c_{1}{\dot{\theta }}_{1}\\ 0 \end{array}\right]{}_{1}^{0}\vec{\omega }=\left[\begin{array}{c} 0\\ 0\\ {\dot{\theta }}_{1} \end{array}\right]$$

The centroid position vector, the centroid velocity vector and the centroid angular velocity vector of the human torso are:

$${}_{2}^{0}\vec{P}=\left[\begin{array}{c} r_{2}c_{1}\\ r_{2}s_{1}\\ 0 \end{array}\right]{}_{2}^{0}\vec{V}=\left[\begin{array}{c} -r_{2}s_{1}{\dot{\theta }}_{1}\\ r_{2}c_{1}{\dot{\theta }}_{1}\\ 0 \end{array}\right]{}_{2}^{0}\vec{\omega }=\left[\begin{array}{c} 0\\ 0\\ {\dot{\theta }}_{1} \end{array}\right]$$

The centroid position vector, the centroid velocity vector and the centroid angular velocity vector of the human upper limb are:

$${}_{3}^{0}\vec{P}=\left[\begin{array}{c} l_{2}c_{1}+r_{3}c\left(\theta_{1}-\theta_{2}\right)\\ l_{2}s_{1}+r_{3}s\left(\theta_{1}-\theta_{2}\right)\\ 0\\ 1 \end{array}\right]{}_{3}^{0}\vec{V}=\left[\begin{array}{c} -l_{2}s_{1}{\dot{\theta }}_{1}-r_{3}s\left(\theta_{1}-\theta_{2}\right)\left({\dot{\theta }}_{1}-{\dot{\theta }}_{2}\right)\\ l_{2}c_{1}{\dot{\theta }}_{1}+r_{3}c\left(\theta_{1}-\theta_{2}\right)\left({\dot{\theta }}_{1}-{\dot{\theta }}_{2}\right)\\ 0 \end{array}\right]{}_{3}^{0}\vec{\omega }=\left[\begin{array}{c} 0\\ 0\\ {\dot{\theta }}_{1}+{\dot{\theta }}_{2} \end{array}\right]$$

The centroid position vector, the centroid velocity vector and the centroid angular velocity vector of the object are:

$${}_{4}^{0}\vec{P}=\left[\begin{array}{c} l_{2}c_{1}+l_{3}c\left(\theta_{1}-\theta_{2}\right)\\ l_{2}s_{1}+l_{3}s\left(\theta_{1}-\theta_{2}\right)\\ 0\\ 1 \end{array}\right]{}_{4}^{0}\vec{V}=\left[\begin{array}{c} -l_{2}s_{1}{\dot{\theta }}_{1}-l_{3}s\left(\theta_{1}-\theta_{2}\right)\left({\dot{\theta }}_{1}-{\dot{\theta }}_{2}\right)\\ l_{2}c_{1}{\dot{\theta }}_{1}+l_{3}c\left(\theta_{1}-\theta_{2}\right)\left({\dot{\theta }}_{1}-{\dot{\theta }}_{2}\right)\\ 0 \end{array}\right]{}_{4}^{0}\vec{\omega }=\left[\begin{array}{c} 0\\ 0\\ {\dot{\theta }}_{1}+{\dot{\theta }}_{2} \end{array}\right]$$

The centroid velocity of the member i(i=1,2,3,4) is $v_{i}$, the moment of inertia around the centroid is $J_{i}$, the plane where o point located is the zero potential energy surface of the gravitational potential energy. If the elasticity between the components and the motion pair are not taken into account, the total kinetic energy *E* and the potential energy *V* of the entire mechanism are [20]:

(8) $$E=\sum_{i=1}^{2}\frac{1}{2}\left(m_{i}v_{i}^{2}+J_{i}{\dot{\theta }}_{i}^{2}\right)=\frac{1}{2}J_{11}{\dot{\theta }}_{1}^{2}+J_{12}{\dot{\theta }}_{1}{\dot{\theta }}_{2}+\frac{1}{2}J_{22}{\dot{\theta }}_{2}^{2}$$

(9) $$V=\sum_{i=1}^{4}m_{i}g{}_{i}^{0}{\vec{P}}_{y}$$

Equation (8) is obtained for $\theta_{1},\theta_{2}$ and ${\dot{\theta }}_{1},{\dot{\theta }}_{4}$ respectively [20]:

(10) $$\begin{array}{c} \frac{\partial E}{\partial \theta_{i}}=\frac{1}{2}\frac{\partial J_{11}}{\partial \theta_{i}}{\dot{\theta }}_{1}^{2}+\frac{\partial J_{12}}{\partial \theta_{i}}{\dot{\theta }}_{1}{\dot{\theta }}_{2}+\frac{1}{2}\frac{\partial J_{22}}{\partial \theta_{i}}{\dot{\theta }}_{2}^{2}\left(i=1,2\right)\\ \frac{\partial E}{\partial {\dot{\theta }}_{i}}=J_{1i}{\dot{\theta }}_{1}+J_{i2}{\dot{\theta }}_{2}\left(i=1,2\right) \end{array}$$

Substituting the Formula (10) into the Lagrange equation:

(11) $$\frac{d}{dt}\left(\frac{\partial E}{\partial {\dot{\theta }}_{i}}\right)-\frac{\partial E}{\partial {\dot{\theta }}_{i}}+\frac{\partial V}{\partial {\dot{\theta }}_{i}}=\tau_{i}$$

Bring two formula above represented as matrix form:

(12) $$\begin{array}{c} \left[\begin{array}{c} T_{1}\\ T_{2} \end{array}\right]=\left[\begin{array}{cc} (m_{1}r_{1}^{2}+I_{12,34}+m_{2}r_{2}^{2}+m_{3}\left(l_{2}^{2}+r_{3}^{2}\right)+m_{4}\left(l_{2}^{2}+l_{3}^{2}\right)+2\left(m_{3}l_{2}r_{3}+m_{4}l_{2}l_{3}c_{2}\right) & \\ -(m_{3}r_{3}^{2}-I_{3}+m_{4}l_{3}^{2}-I_{4}+\left(m_{3}l_{2}r_{3}+m_{4}l_{2}l_{3}\right)c_{2}) & \end{array}\right]\left[\begin{array}{c} {\ddot{\theta }}_{1}\\ {\ddot{\theta }}_{2} \end{array}\right]\\ +\left[\begin{array}{c} -(m_{3}r_{3}^{2}-I_{3}+m_{4}l_{3}^{2}-I_{4}+\left(m_{3}l_{2}r_{3}+m_{4}l_{2}l_{3}c_{2}\right)\\ m_{3}r_{3}^{2}+I_{3}+m_{4}l_{3}^{2}+I_{4} \end{array}\right]\left[\begin{array}{c} {\ddot{\theta }}_{1}\\ {\ddot{\theta }}_{2} \end{array}\right]\\ +\left[\begin{array}{cc} 0 & \left(m_{3}r_{3}+m_{4}l_{3}\right)l_{2}s_{2}\\ -\left(m_{3}r_{3}+m_{4}l_{3}\right)l_{2}s_{2} & 0 \end{array}\right]\left[\begin{array}{c} {\dot{\theta }}_{1}^{2}\\ {\dot{\theta }}_{2}^{2} \end{array}\right]+\left[\begin{array}{cc} -2\left(m_{3}l_{2}r_{3}+m_{4}l_{2}l_{3}\right)s_{2} & 0\\ 2\left(m_{3}l_{2}r_{3}+m_{4}l_{2}l_{3}\right)s_{2} & 0 \end{array}\right]\left[\begin{array}{c} {\dot{\theta }}_{1}{\dot{\theta }}_{2}\\ {\dot{\theta }}_{2}{\dot{\theta }}_{1} \end{array}\right]\\ +\left[\begin{array}{c} \left(m_{1}r_{1}+m_{2}r_{2}+m_{3}l_{2}+m_{4}l_{2}\right)gc_{1}+\left(m_{3}r_{3}+m_{4}l_{3}\right)gc\left(\theta_{1}-\theta_{2}\right)\\ \left(m_{3}gr_{3}+m_{4}gl_{3}\right)c\left(\theta_{1}-\theta_{2}\right) \end{array}\right] \end{array}$$

During the process of lifting heavy objects, the objects are always vertically downwards, the geometry is $\theta_{2}=\frac{\pi }{2}+\theta_{1}$. Substituting into the above Formula (12):

$$\begin{array}{c} \left[T\right]=\left[m_{1}r_{1}^{2}+I_{1}+m_{2}r_{2}^{2}+I_{2}+m_{3}l_{2}^{2}+m_{4}l_{2}^{2}+4\left(I_{3}+I_{4}\right)\right]\left[{\ddot{\theta }}_{1}\right]\\ +\left[\left(m_{1}gr_{1}+m_{2}gr_{2}+m_{3}gl_{2}+m_{4}gl_{2}\right)\cos\theta_{1}\right] \end{array}$$

### 3.6. Control Strategy

The handling operation means that the user lifts the heavy objects from the stoop position to the upright position with the assist of the exoskeleton [23]. Because the size of lifting objects and the users’ height are different, each person has a different starting position when lifting a heavy object, the initial angle is obtained according to angle sensor, and the end position is an upright state. The motion trajectory of the exoskeleton from the initial pose to the final pose must be smooth. In order to avoid impact, the speed and acceleration of the exoskeleton system at the start and end times should be also 0. The motion trajectory curve in this paper is a 5th degree polynomial function. The specific motion rules are as follows:

(13) $$\theta \left(t\right)=a_{1}t^{5}+b_{1}t^{4}+c_{1}t^{3}+d_{1}t^{2}+e_{1}t+f_{1}$$

where $\theta_{0}$ is acquired by angle sensor in real time, $\theta_{t_{f}}\;=\;90^{\circ }$, ${\dot{\theta }}_{t=t_{0}}=0$, ${\dot{\theta }}_{t=t_{f}}=0$, ${\ddot{\theta }}_{t=t_{0}}=0$, ${\ddot{\theta }}_{t=t_{f}}=0$. 

**Figure 11:**
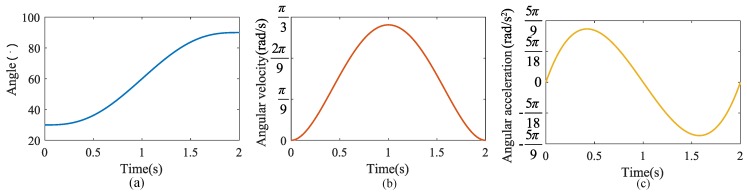
The planning trajectory. (**a**) angle, (**b**) angular velocity, (**c**) angular acceleration.

The planning trajectory results are shown in Figure 11. Figure 11a is the angle curve, the initial position is set to $30^{\circ }$. Figure 11b,c are the angular velocities and angular acceleration curve respectively. The velocity and acceleration at the initial and end times are both 0, which ensures a smooth transition at the start and the end of lifting task, avoiding rigid impact and reducing damage to the motor.

Without considering friction and other disturbances, the dynamic equation of the exoskeleton can be obtained according to front dynamic equation:

(14) $$H\left(q\right)\ddot{q}+C\left(q,\dot{q}\right)\dot{q}+G\left(q\right)=T$$

where *q* is the joint dimension, H(q) is the robot inertia matrix, $C(q,\dot{q})$ is the centripetal force and Coriolis force, G(q) is the moment formed by gravity, and *T* is the torque or force applied to each joint. Set the expected amount of *q* to $q_{d}$, which is the expected trajectory of the robot.

In the assist process of the waist exoskeleton, the adaptive controller provides a n×1 vector of assist torque *T* at each joint. *T* can be determined by a model-based adaptive control algorithm in paper [24]. Defined as:

(15) $$T=H\left(q\right){\ddot{q}}_{d}+C\left(q,\dot{q}\right){\ddot{q}}_{d}+G\left(q\right)-K_{D}\dot{\tilde{q}}-K_{P}\tilde{q}$$

where: ${\tilde{q}}_{\left(t\right)}=q_{\left(t\right)}-{q_{d}}_{\left(t\right)}$ is the trajectory tracking error, $q_{d}$ is the exoskeleton planning trajectory. $K_{D},K_{P}$ are the time-varying positive definite matrix.

If it is limited to a sliding surface $\dot{\tilde{q}}+\Lambda \tilde{q}=0$, an unnecessary steady-state position error fraction can be eliminated, and Λ is a constant matrix whose eigenvalue are located on the right half-complex plane, and $\Lambda =K_{D}\cdot K_{P}^{-1}$.

Introduce a new quantity $q_{r}$ as a virtual reference trajectory.

(16) $${\dot{q}}_{r}={\dot{q}}_{d}-\Lambda \tilde{q},{\ddot{q}}_{r}={\dot{q}}_{d}-\Lambda \dot{\tilde{}}q$$

Therefore, the control strategy can be rewritten as:

(17) $$T=\hat{H}\left(q\right)\cdot {\ddot{q}}_{r}+\hat{C}\left(q,\dot{q}\right)\cdot {\dot{q}}_{r}+\hat{G}\left(q\right)-K_{D}\cdot s$$

Definition $\tilde{a}$ is the the system estimation error vector $\tilde{a}=\hat{a}-a$.

(18) $$\dot{\hat{a}}=-\Gamma^{-1}Y^{T}\left(\dot{\tilde{q}}+\Lambda \tilde{q}\right)=-\Gamma^{-1}Y^{T}S$$

where Γ is the n×n order positive definite gain matrix, which determines the controller’s adaptive rate based on the overall error. In the literature [25], the regression matrix *Y* is determined for adapting to different people by using RBF (Gaussian radial basis function). By selecting the *Y* parameter vector in this way, *a* becomes a feed-forward term that determines the robot drive torque *T*,

(19) $$Y=\exp(\frac{{\left|q-q_{d}\right|}^{2}}{2\sigma^{2}})$$

A quantity $-\frac{1}{\tau }Y^{T}{\left(YY^{T}\right)}^{-1}Y\hat{a}$ of “assisted-as-needed” is introduced in the standard adaptive strategy [25] to limit the rate of change of the parameter *a* to prevent the parameter estimator $\hat{a}$ from having a large influence on the robot output torque in the time constant τ. The adaptive control strategy can be written as:

(20) $$\dot{\hat{a}}=-\frac{1}{\tau }Y^{T}{\left(YY^{T}\right)}^{-1}Y\hat{a}-\Gamma^{-1}Y^{T}S$$

**Figure 12:**
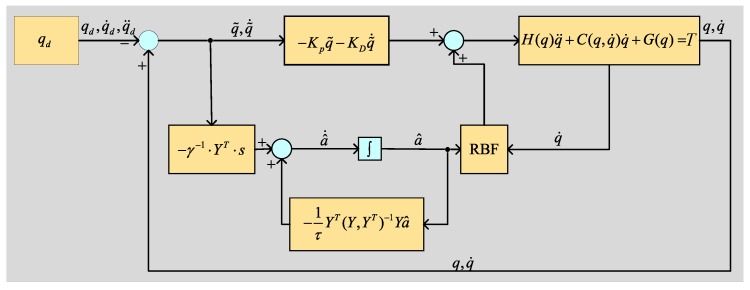
A block diagram of the control.

The global stability proof of the algorithm has a detailed derivation process in the literature [25]. The block diagram of the control shows in Figure 12.

## 4. Experiment

**Figure 13:**
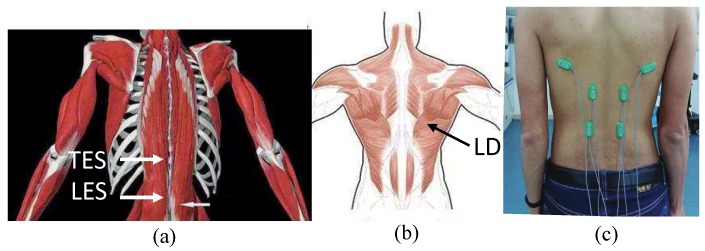
(**a**) shows the erector spinae of the LES and TES, (**b**) depicts the LD, (**c**) displays the electrode position on a subject’s back.

There are more than 29 muscles of human back involved in lifting [26]. The erector spinae is one of the most important muscles that allows the spines to erect. It is located on both sides of the spine and connected the head as well as the tail-bone. It mainly consists of the spine and the longissimus. Clinical studies have found that most low back pain is caused by vertebral muscle strain [27]. The erector spinae play a major role in the process of bending and erecting. The activity of the muscle can be reflected by the EMG amplitude, thus the erector spinae is selected as an assessment target. The erector spinae includes the LES, the TES and the LD, as shown in the Figure 13a,b.

### 4.1. Participants

Ten subjects who had no history of lumbar disease or spinal disease volunteered to participate in the study (average age of 26 years, weight of 70 kg, and height of 174 cm). These subjects were asked to read experimental protocol and signed a consent form prior to the trial. This test was performed according to the Research Ethics Procedures of the Shenzhen Institutes of Advanced Technology.

### 4.2. Testing Equipment and Surface Electromyography

The myoelectric equipment used to record electromyographic signals was Biometrics Ltd. (Sampling rate: 1000 Hz). It has 8 channels and one reference electrode. The small wireless adapter connects to the USB port of a PC host and data input is through a WIFI communication. A standard storage box (46 mm × 56 mm × 30 mm) with hand-holes was placed in front of the subject. Six different loads (0, 5, 10, 15, 20, and 25 kg) were used in this trial. The loads studied reflect a range from low to high in industrial tasks [17].

EMG signals of three muscles on the left and right side of the back was studied. The recording sites were: the lumbar erector spinae (LES) at the level of L3 vertebrae with interval of 4 cm, the thoracic erector spinae (TES) at the level of T9 vertebrae with interval 4 cm, and latissimus dorsi [28]. The reference electrode was placed on the elbow joint. Before the EMG electrodes were fixed at the above positions, the skin was cleaned with alcohol cotton ball. Figure 13c shows the electrode positions on a subject’s back.

### 4.3. Testing Procedures

**Figure 14:**
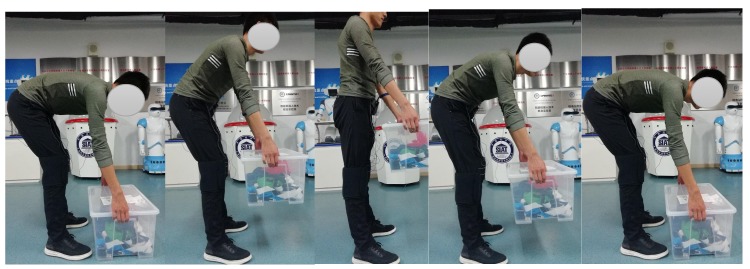
The process of lifting box.

Before starting each test, subjects were informed of the detailed testing procedure and setup of exoskeletons, then we adjusted the leg length of the exoskeleton according to the subject’s body size and verified the connection of the data acquisition system. Subjects were required to practice the testing procedure initially. Testing began once subjects were proficient and comfortable with the lifting tasks. The subjects maintained a standing posture at the beginning, then began to slowly bend forward and lifted the box in a stooped posture. After lifting the box, the subject’s legs were upright and they held the weight for 8–10 s. The subject then slowly lowered the box on the ground and resumed the upright posture. The process of lifting the box is shown in Figure 14. Participates were asked to performed five cycles for each load, with at least 1 min rest period between each cycle.

## 5. Results

Ten subjects participated in the trial. Lower back muscle EMG signals of the left and right LES, TES and LD were recorded in symmetrical lifting for six different objects (0, 5, 10, 15, 20, and 25 kg) under two experimental conditions, with and without the waist assist exoskeleton. After filtering with a bandwidth of 10–500 Hz [29] and other processing of EMG signals [30], calculating their integral IEMG values within a lifting cycle, we defined the assistance efficiency of the exoskeleton by the following formula:

$$E=1-\frac{I_{E}}{I}$$

where $I_{E}$ is the IEMG when the subjects lift the loads with the exoskeleton, and *I* is the IEMG when the subjects lift the same loads without the exoskeleton.

**Figure 15:**
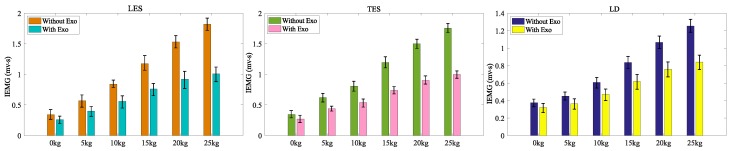
IEMG of LES, TES and LD when lifting 0–25 kg objects with and without exoskeleton.

The EMG signals of the left and right muscles were not significantly different in accordance with initial statistical analyses, therefor the EMG data of left and right muscles were pooled and then averaged for each of the ten participates. Figure 15 shows the average IEMG of LES, TES and LD for lifting 0 kg to 25 kg loads respectively under the two conditions, with and without the exoskeleton. Table 2 displays the statistical test results. The waist assist exoskeleton significantly reduced the activities of the LES, TES and LD. When wearing the waist exoskeleton, the amount of IEMG reduction ranged from 23.5 ± 5.5% to 44.5 ± 6.5% for the LES, and the amount of IEMG reduction ranged from 20.5 ± 4.5% to 42.8 ± 6.6% for TES when lifting 0 kg to 25 kg, respectively. The lower-back muscle LD was increased from 13.5 ± 2.8% to 32.8 ± 5.8% when wearing the exoskeleton for the 0 kg to 25 kg lifting task. The average integrated electromyography reductions were 34.0%, 33.9% and 24.1% for the TES, LES and LD respectively. From the Table 2, we can see that the LES and TES decreased by larger rates than the LD when lifting the same objects with the SIAT waist exoskeleton. As the weights of the lifted objects increase, the muscle activity of the LES, the TES and the LD also gradually increased. When the weight of the object is 25 kg, the decrease in the rates of LES, TES and LD reach 44.5 ± 6.5%, 42.8 ± 6.6%, 32.8 ± 5.8% respectively. The experimental results demonstrate that the SIAT waist exoskeleton can reduce muscle fatigue during lifting tasks. It can help to reduce the burden and back pain, and the incidence of lumbar muscle strain during long-term lifting work.

**Figure 16:**
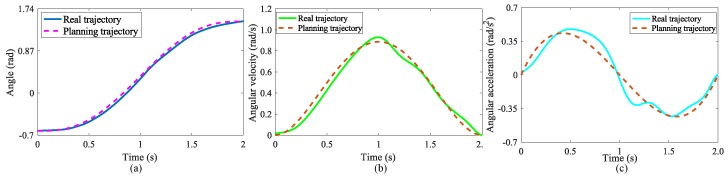
The comparison between real trajectory and planning. (**a**) angle, (**b**) angular velocity, (**c**) angular acceleration.

Figure 16a shows the comparison between the average of real angle and planning. The exoskeleton can generate smooth trajectory with angles obtained from different users’ initial postures, and the motors complete movements based on proposed control algorithm. Figure 16b,c show the comparison between real angular velocity, real angular acceleration and planning respectively. It can be seen that the values of the real angular velocity and real angular acceleration at the beginning and end of the curve are both 0, which are very close to the theoretical value. The comparison results demonstrate that the motors movement trajectories are able to meet the theoretical requirement based on the adaptive control algorithm, which can avoid the impact during the motion process.

## 6. Conclusions and Discussion

The purpose of this paper was to describe the design and control algorithm of a powered waist assist exoskeleton that has been specially developed for industrial material handling. The assessment of the potential effects of the device to reduce physical loading on the lower back based on IEMG was also described in this paper. The exoskeleton has active hip joints to provide external assistance for flexion/extension during lifting and reduce the risk of developing WMSD.

Compared with other active waist exoskeletons designed for providing assistances [10,11,12,13], the proposed exoskeleton has several structural advantages. First, we optimized the exoskeleton’s mechanical structure to make it convenient to employ for many applications. We developed an ergonomic design to improve the fit for the human lumbar vertebrae joints. The device is easy to put on and requires no additional accessories. The total system weights only 5 kg, making it easily portable. Furthermore, to prevent unnecessary power consumption, we installed a clutch on the exoskeleton. Finally, the SIAT waist exoskeleton automatically detects the users’ intended motions when lifting objects using IMU.

The exoskeleton can generate smooth trajectories with angles obtained from different users’ initial postures by angle sensors in real time, and the motors then complete movements based on an adaptive control algorithm. When wearing the SIAT waist exoskeleton, the measured myoelectric signals of the lumbar erector spinae (LES), thoracic erector spinae (TES) and latissimus dorsi (LD) of the user’s back decreased by 44.5 ± 6.5%, 42.8 ± 6.6% and 32.8 ± 5.8% during lifting 25 kg respectively. The experimental results indicate that wearing the device can reduce muscle fatigue of the erector spinae and latissimus dorsi when lifting heavy objects.

There are some limitations of the exoskeleton proposed in this study. First, this device cannot provide assist when lowering heavy objects. Thus, additional structural designs are needed to allow the exoskeleton to provide mechanical support when lowering objects. Second, this SIAT waist exoskeleton is controlled by tracking the planned trajectories without fully considering the interaction between the users and the exoskeleton. Novel control strategies should be considered to make the exoskeleton more adaptive in the future. Third, our experiments were limited to the laboratory. Additional experimental tests should be conducted by workers engaged in material handling in actual industrial applications. Further exploration in mechanical design, modeling, and motor control are needed to make the SIAT waist exoskeleton more applicable for workers when lifting and holding heavy objects in industry, thereby improving quality of life.

## Figures and Tables

**Table 1:** Framework of identified industrial exoskeletons.

Exoskeleton	Tasks	Power Supply	Weight (kg)	Battery Life (h)	Loading (kg)
ATOUN Model A	B, L	Servo motor	6.7	2.5	25
ATOUN Model AS	B, L, C	Servo motor	6.7	2.5	25
ATOUN Model Y	B, L, C	Servo motor	4.5	8	25
HAL Lumbar	B, L	Hydraulic actuators	3	-	7.5
Muscle Suit	L, H	Artificial muscle	5.5	Gas supply	35
Buddy	B, L	Servo motor	6.8	3	13
SIAT waist EXO	B, L	Servo motor	5	4	25

EXO—exoskeleton, B—bending, L—lifting, C—carrying, H—holding.

**Table 2:** The statistical data of IEMG and the assistance efficiency of exoskeleton under different loads.

	0 kg	5 kg	10 kg	15 kg	20 kg	25 kg
LES						
Without Exo (mv·s)	0.34 ± 0.01	0.56 ± 0.02	0.84 ± 0.03	1.17 ± 0.05	1.52 ± 0.06	1.82 ± 0.06
With EXo (mv·s)	0.26 ± 0.01	0.40 ± 0.01	0.562 ± 0.02	0.76 ± 0.03	0.922 ± 0.03	1.01 ± 0.03
Efficiency (%)	23.5 ± 5.5	28.6 ± 7.1	33.3 ± 6.9	35 ± 7.6	39.3 ± 7.2	44.5 ± 6.5
TES						
Without Exo (mv·s)	0.34 ± 0.01	0.62 ± 0.02	0.81 ± 0.03	1.19 ± 0.04	1.5 ± 0.05	1.75 ± 0.06
With EXo (mv·s)	0.27 ± 0.01	0.44 ± 0.01	0.54 ± 0.02	0.74 ± 0.02	0.9 ± 0.02	1 ± 0.03
Efficiency (%)	20.5 ± 4.5	29.0 ± 6.3	33.3 ± 6.6	37.8 ± 6.5	40 ± 5.8	42.8 ± 6.6
LD						
Without Exo (mv·s)	0.37 ± 0.01	0.45 ± 0.01	0.61 ± 0.01	0.84 ± 0.03	1.07 ± 0.03	1.25 ± 0.03
With EXo (mv·s)	0.32 ± 0.01	0.36 ± 0.01	0.47 ± 0.01	0.62 ± 0.02	0.76 ± 0.02	0.84 ± 0.02
Efficiency (%)	13.5 ± 2.8	20.0 ± 3.2	22.9 ± 4	26.2 ± 5.7	29 ± 6	32.8 ± 5.8
